# Effect of a Peer Support Intervention for Oral Pre‐Exposure Prophylaxis Uptake and Adherence Among Adolescent Girls and Young Women at Risk of HIV Acquisition in Kampala, Uganda: A Randomized Controlled Trial

**DOI:** 10.1002/jia2.70210

**Published:** 2026-09-21

**Authors:** Yunia Mayanja, Andrew Abaasa, Kenneth Roger Katumba, Matthew Spinelli, Bernard Kikaire, Monica Gandhi, Kyriaki Kosidou, Anna Mia Ekstrom, Eugene Ruzagira

**Affiliations:** ^1^ Medical Research Council/Uganda Virus Research Institute and London School of Hygiene and Tropical Medicine (MRC/UVRI & LSHTM) Uganda Research Unit Entebbe Uganda; ^2^ Department of Global Public Health Karolinska Institutet Stockholm Sweden; ^3^ London School of Hygiene and Tropical Medicine London UK; ^4^ Division of HIV, Infectious Diseases and Global Medicine, Department of Medicine University of California San Francisco San Francisco California USA; ^5^ Uganda Virus Research Institute Entebbe Uganda; ^6^ Department of Paediatrics College of Health Sciences Makerere University Mulago Uganda; ^7^ Centre for Epidemiology and Community Medicine Stockholm Sweden; ^8^ Department of Infectious Diseases/Vithusan Södersjukhuset Sweden; ^9^ Department of Clinical Science and Education Södersjukhuset Sweden

**Keywords:** adherence, adolescent girls and young women (AGYW), oral pre‐exposure prophylaxis (PrEP), peer support/peer education, randomized controlled trial, Uganda

## Abstract

**Introduction:**

Daily oral pre‐exposure prophylaxis (PrEP) use remains low among adolescent girls and young women (AGYW). We evaluated whether a peer support intervention improved daily oral PrEP uptake and adherence among AGYW at high risk of HIV acquisition in Kampala, Uganda.

**Methods:**

We conducted an open‐label randomized controlled trial (January 2023−June 2024) among 14‐ to 24‐year‐olds engaged in sex work. Eligible participants were randomized 1:1 to receive either health worker counselling only (control) or supplemented with peer support (intervention). Peer leaders delivered co‐created peer education sessions starting 1 month after enrolment. Participants were followed for 12 months. Co‐primary outcomes were PrEP uptake (yes/no) and adherence, evaluated using two separate measures; optimal if: ≥85% pill‐counts (primary measure); positive point‐of‐care urine tenofovir test. Both arms received adherence counselling based on urine results. Secondary outcomes: PrEP persistence (time to discontinuation) and cost‐effectiveness (cost per disability adjusted life year/DALY averted). Analysis used logistic regression (uptake); generalized estimating equations (adherence), Cox regression (persistence) and incremental cost‐effectiveness ratio (ICER) below US$537 per DALY averted was considered cost‐effective.

**Results:**

We enrolled 394 participants (197 per arm) with a mean age of 19.4 (SD ±2.3) years. The intervention did not impact PrEP uptake (OR 1.15; 95% CI 0.77−1.71) but improved adherence, pill count (OR 2.20; 95% CI 1.23−3.95) and urine assay (OR 2.18; 95% CI 1.22−3.91). Optimal adherence was low but significantly higher in the intervention by both measures: pill count (intervention = 54.8%, control = 39.6%, *p* = 0.008), urine assay (intervention = 54.8%, control = 40.8%, *p* = 0.001). Median duration before PrEP discontinuation was 38 days (IQR 0–319 days); intervention (56 days), control (29 days). The intervention had no effect on persistence (HR 0.86; 95% CI 0.64−1.14). It was cost‐effective with the urine tenofovir assay (ICER = $459) but not with pill counts (ICER = $581).

**Conclusions:**

This peer‐led intervention improved PrEP adherence among AGYW in Kampala and is recommended in high‐incidence settings. Urine tenofovir testing can be used to assess adherence if affordable. Enhanced peer support strategies are needed until long‐acting PrEP becomes accessible and acceptable. Future research should optimize timing, intensity and delivery models of peer support interventions and integrate them with long‐acting PrEP to improve sustainability.

## 1 Introduction

Sub‐Saharan Africa (SSA) accounted for 50% of the 1.3 million new HIV acquisitions in 2024. Adolescent girls and young women (AGYW) aged 15–24 years in the region contributed 83% of weekly global acquisitions in this group [1]. In SSA, AGYW are three times more likely to acquire HIV than their male peers [2]. Although multiple pre‐exposure prophylaxis (PrEP) options are recommended, including daily oral PrEP, the dapivirine vaginal ring and injectable cabotegravir and twice‐yearly injectable lenacapavir [3, 4, 5, 6], daily oral PrEP remains the most accessible option in SSA despite varying AGYW preferences [7, 8, 9].

Oral PrEP uptake remains low (22%–37%) among AGYW at risk of HIV acquisition [10, 11, 12], with low adherence (<45%) and persistence (37%–56%) [11, 12, 13], due to multi‐level barriers [10, 14, 15]. Interventions targeting PrEP uptake and adherence have primarily been health worker‐delivered, including pharmacy‐based services [16], support clubs, electronic monitoring and mobile‐health strategies [17, 18, 19], with mixed adherence effects. While retrospective drug‐level feedback using dry blood spots has not improved adherence among AGYW [13], immediate feedback using point‐of‐care urine tenofovir testing improved adherence [20, 21]. Furthermore, combining health worker engagement with peer support improves adherence [19].

Peer‐led strategies are feasible and acceptable [22, 23, 24], but have shown mixed effects on linkage to HIV services and PrEP uptake [23, 25, 26]. Enhanced peer referral is promising as it involves training peer leaders; however, combining it with HIV self‐test delivery did not improve PrEP uptake or persistence among AGYW [24]. Nonetheless, peer networks facilitate PrEP use [22, 27, 28], while combined health worker–peer support packages have improved adherence [19]. Peer‐led interventions vary in design and delivery; however, young people often adopt peer behaviours within groups [29]. We evaluated a co‐created group‐based peer support intervention aimed at improving oral PrEP uptake and adherence among AGYW vulnerable to HIV acquisition in Kampala, Uganda.

## 2 Methods

### 2.1 Study Design and Setting

From January 2023 to June 2024, we conducted an open‐label randomized controlled trial (RCT), “Peers for PrEP” (NCT05516602). Participants were randomized 1:1 to either standard care /health‐worker counselling (control arm) or standard care plus peer support (intervention arm) using permuted blocks (sizes 8, 10, 12). Allocation was concealed through sequentially numbered opaque, sealed envelopes. We hypothesized that peer support through peer education alongside health worker counselling would improve PrEP knowledge and attitudes, thereby increasing uptake and adherence. The study was conducted at the AIDS Information Centre (AIC), a non‐governmental organization providing HIV‐related services, including oral PrEP supported by the U.S. President's Emergency Plan for AIDS Relief (PEPFAR). Participants accessed free HIV prevention and reproductive health.

### 2.2 Study Population and Recruitment

Field workers and community mobilizers mapped 20 Kampala communities (northern: 9, southern: 8, central: 3) known for entertainment and sex work. Mobilizers referred AGYW to AIC for screening and enrolment and received UGX 30,000 (∼US$8) per 15 enrolments. Eligibility required being HIV and hepatitis B negative, 14–24 years, sexually active in the previous quarter and willing to attend visits. Exclusion included HIV acquisition, pregnancy, medical/psychosocial barriers to consent or participation, or current PrEP use.

### 2.3 Intervention Development and Implementation

Peer leader selection, training and intervention co‐creation are described elsewhere [30]. Briefly, eight youths aged 18–24 years who had initiated daily oral PrEP or antiretroviral therapy (ART) for ≥6 months co‐created the intervention with study counsellors through three iterative meetings to identify and refine intervention components. Delivery was led by two peer leader groups: (i) four females using PrEP and (ii) four using ART (two females, two males). The final intervention was standardized in a script to ensure fidelity and comprised five components involving both information delivery and interactive activities (Table 1).

**TABLE 1:** Peer education intervention components that were delivered to AGYW by peer leaders, Kampala, Uganda (February−October 2023).

(i)	Education on HIV risk reduction, oral PrEP *(benefits of PrEP, side effects, addressing stigma, strategies for disclosure of PrEP use, distinguishing PrEP from antiretroviral therapy)*
(ii)	Dispelling myths and misconceptions about PrEP
(iii)	Peer leaders sharing personal experiences with daily pill use and coping strategies
(iv)	AGYW sharing their experiences and challenges with PrEP with peer leaders facilitating support discussions
(v)	1−2 role plays per session followed by feedback and discussions of lessons learned *(Role play themes picked from topics in the first component)*

Abbreviations: AGYW, adolescent girls and young women; PrEP, pre‐exposure prophylaxis.

*Source*: Reproduced from Mayanja et al., 2025, Frontiers in Public health, CC BY 4.0.

Two experienced counsellors trained peer leaders in peer education, intervention delivery and HIV prevention for lay providers per national PrEP guidelines [31]. Six of AGYW groups (20–40 participants each) were randomly assigned to peer leaders on PrEP (three groups) or ART (three groups). The five intervention components were delivered in‐person over 2‐ to 2.5‐h per session. Each participant received one session monthly for 4 months. Sessions began 1‐month post‐enrolment to encourage PrEP initiation among those not initiated after health worker counselling, and to support adherence. Field workers made reminder calls, and peer leaders coordinated session schedules.

### 2.4 Sample Size

Sample sizes (PrEP uptake and adherence) were calculated (Stata v18.0) to achieve 80% power with a two‐sided 95% confidence interval, allowing 10% loss to follow‐up. Assuming 31% uptake with health‐worker counselling [12] and a 16% increase with peer support [32], and 33% high adherence with a projected 20% increase [27], required sample sizes were 314 (uptake) and 210 (adherence). The larger sample was considered. After a 25.6% month‐3 missed visit rate (18.3% intervention; 33.0% control), ethical approval to enrol 80 (25%) additional participants was obtained, yielding a final sample of 394.

#### 2.4.1 Study Arms

##### 2.4.1.1 Control

Individualized health worker counselling during weekday clinic visits delivered by four staff (three study nurses, one counsellor).

##### 2.4.1.2 Intervention

Like control, plus peer‐led group education on Saturdays, a non‐clinic day reserved for the intervention group and aligned with peer leaders’ availability.

### 2.5 Study Procedures

Participants were followed quarterly for 12 months. PrEP initiators received a 1‐month supply of tenofovir disoproxil fumarate (300 mg)/emtricitabine (200 mg), then 1–3 month refills based on adherence and preference. Return of leftover pills was encouraged for pill‐count adherence assessments. In both arms, study nurses conducted point‐of‐care urine tenofovir testing with real‐time feedback and adherence counselling. Field workers made reminder calls and, with mobilizers, traced unreachable participants. Participants received UGX 35,000 (∼US$9) per visit. When clinic attendance was impossible, field‐based data and sample collection was conducted. Individuals diagnosed with HIV were withdrawn and referred for treatment.

### 2.6 Data Collection

Following training, participants completed a quarterly tablet‐based REDCap questionnaire in Luganda and English with text, and audio option for low‐literacy participants. It assessed sexual behaviour, reproductive health, intimate partner violence and drug use. Study nurses documented socio‐demographics, PrEP uptake and discontinuation dates, pill counts and laboratory results on paper forms. Data were double‐entered into REDCap after quality checks.

Samples collected included: serum for hepatitis B screening at enrolment (Abbott HBsAg), quarterly HIV testing (national algorithm) and creatinine at PrEP initiation (Roche CREP2); plasma for HIV drug resistance at seroconversion; and urine for tenofovir levels at PrEP initiation and refills using a point‐of‐care assay (Abbott/Alere) [33], quarterly chlamydia/gonorrhoea (GeneXpert) and pregnancy (Pal βHCG) testing.

### 2.7 Study Outcomes

#### 2.7.1 Primary Outcomes

- PrEP uptake: first primary outcome defined as accepting oral PrEP and collecting the initial 1‐month supply at any time during the study, documented by study staff, and categorized as binary (yes/no).
- PrEP adherence: second primary outcome assessed quarterly for PrEP initiators via *pill counts*, per national guidelines [31]. For participants with refills between quarterly visits, all data were used to estimate adherence over the quarter. Adherence for a once‐daily regimen was calculated using the formula:
$$\begin{array}{c} \mathrm{Adherence}\;=\frac{\left(\mathrm{dispensed}\;\mathrm{pills}-\mathrm{returned}\;\mathrm{pills}\right)}{\mathrm{number}\;\mathrm{of}\;\mathrm{days}\;\mathrm{since}\;\mathrm{last}\;\mathrm{refill}}\times 100 \end{array}$$

A cut‐off of≥85% [34] categorized adherence as optimal, with values <85% considered suboptimal.

To inform future scale‐up, we also assessed *urine tenofovir levels* (recent PrEP intake), categorized as optimal (positive test: pill taken within past 4 days) or suboptimal (negative test: no pill taken within past 4 days).

#### 2.7.2 Secondary Outcomes

- PrEP persistence: defined as the duration from the PrEP uptake date to the earliest date of (a) first recorded PrEP discontinuation, (b) HIV diagnosis or (c) study exit. Discontinuation was either self‐reported or considered as the end date of a refill period if participants had not returned by the next quarterly assessment.
- Intervention cost‐effectiveness: assessed using the incremental cost‐effectiveness ratio (ICER), defined as:
$$\mathrm{ICER}\;=\frac{\mathrm{Incremental}\;\mathrm{cost}\;\mathrm{of}\;\mathrm{providing}\;\mathrm{the}\;\mathrm{intervention}}{\mathrm{Disability}\;\mathrm{adjusted}\;\mathrm{life}\;\mathrm{years}\;\left(\mathrm{DALYs}\right)\;\mathrm{averted}}$$

##### 2.7.2.1 Contamination

Study nurses asked participants at quarterly visits whether they had discussed peer session activities, that is either told peers (intervention arm) or were told by peers (control arm).

### 2.8 Statistical Analysis

Analyses were conducted in STATA 18.0 (Stata Corp, TX). Participant characteristics were summarized using frequencies and percentages (categorical variables) and means with standard deviations (SD) or medians with interquartile ranges (IQR) (continuous variables). Agreement between pill counts and urine tenofovir assays was evaluated using the kappa statistic.

#### 2.8.1 Primary Analyses

##### 2.8.1.1 PrEP Uptake

Analyses followed an intention‐to‐treat (ITT) approach, including all randomized participants (*N* = 394). Logistic regression estimated the intervention effects, presented as odds ratios (OR) with 95% confidence intervals (CI), and two‐sided *p*‐values.

##### 2.8.1.2 PrEP Adherence

Analyses used a modified ITT approach, including all PrEP initiators who contributed ≥1 refill visit for adherence assessments. Generalized estimating equations with robust standard errors accounted for within‐participant correlation, and estimated population‐averaged effects (ORs, 95% CI and two‐sided *p*‐values). Given the high discontinuation, time interaction was not modelled; however, overall time effects were evaluated using a Wald chi‐square test.

Sensitivity analyses excluded (i) intervention‐arm participants who missed all peer sessions, (ii) potentially contaminated control‐arm participants and (iii) considered those without follow‐up adherence assessment as non‐adherent and contributing a single observation. Adherence by peer leader type was evaluated. We specified two co‐primary outcomes; hence controlled the family‐wise Type I error rate using the Bonferroni correction (97.5%CI, two‐sided *p*<0.025) and applied Holm's method for sensitivity.

#### 2.8.2 Secondary Analyses

##### 2.8.2.1 PrEP Persistence

Cox proportional hazards regression estimated the effect of the intervention on time to PrEP discontinuation, reporting hazard ratios (HR) with 95% CIs and two‐sided *p*‐values. Kaplan−Meier curves and log‐rank tests compared discontinuation between arms.

##### 2.8.2.2 Intervention Cost‐Effectiveness

Analyses included participants attending ≥1 peer‐support session. Costs included peer leader training and allowances, extracted from payment records, excluding research‐related reimbursements not reflective of real‐world PrEP implementation in Uganda. Costs were recorded in Ugandan shillings (December 2022−September 2023), and converted to US dollars using the average exchange rate [35]. Participants were considered adherent only if optimal adherence was achieved at all visits.

DALYs averted per arm were estimated by multiplying the ART‐related DALY weight and expected HIV acquisitions. The standard HIV‐prevention DALY weight comprises life expectancy in the ART era [36], average age at acquisition and HIV incidence [37, 38]. We applied a DALY weight (9.6) considering 22 years as the median age at HIV acquisition among young female sex workers (FSWs) [39] and Ugandan female life expectancy of 70.1 years [40]. Expected HIV acquisitions were based on an incidence of 2.34/100 person‐years among AGYW (oral PrEP arm of the HPTN‐084 trial) [41], adjusted for 90% PrEP efficacy [42]. DALYs were estimated by applying the DALY weight to expected acquisitions. ICER was calculated as cost per DALY averted, with <$537 considered cost‐effective in low‐and middle‐income countries [43].

### 2.9 Ethics

Ethical approval was obtained from the Uganda Virus Research Institute‐Research Ethics Committee (GC/127/918), Uganda National Council for Science and Technology (HS2490ES), the Swedish Ethical Review Authority (Dnr 2023‐05028‐01), and the London School of Hygiene and Tropical Medicine Research Ethics Committee (28193). The Uganda National Drug Authority (NDA/SCI01/65404/2022, NDA/SCI01/78387/2023) approved tenofovir kit importation. AIC gave administrative clearance. Written informed consent was obtained from all participants prior to study procedures, including emancipated and/or mature minors aged ≤17 years.

## 3 Results

### 3.1 Screening, Enrolment and Participant Flow

**FIGURE 1:**
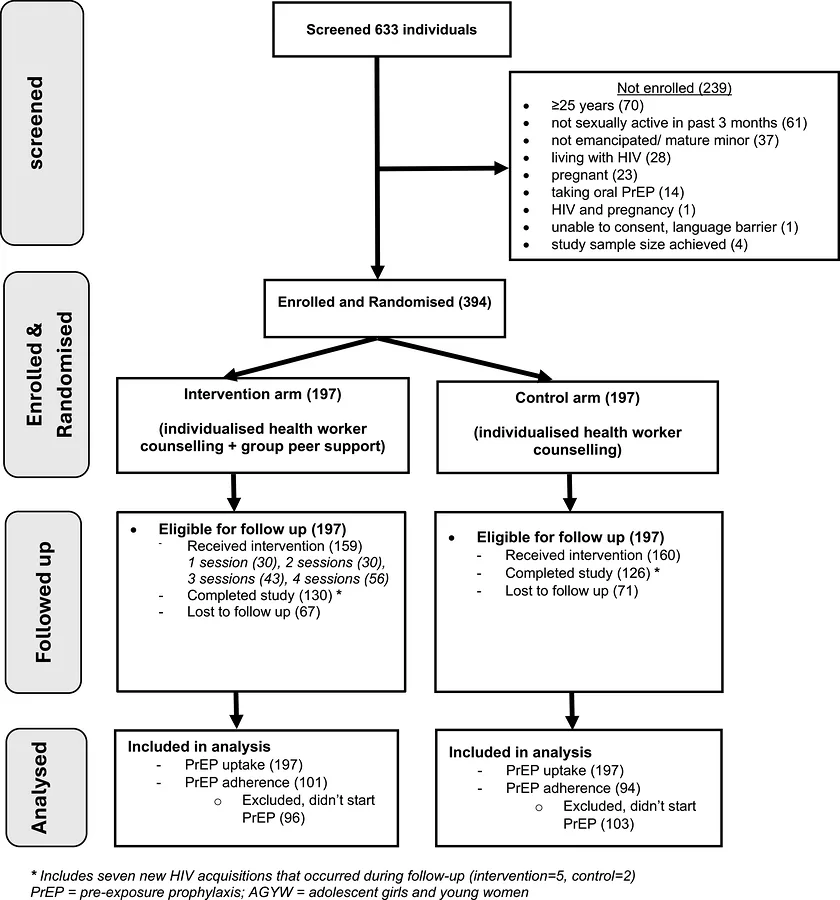
Participant flow of 394 AGYW screened and enrolled in an RCT aimed at assessing the effect of peer support on oral PrEP uptake and adherence, Kampala, Uganda (2023−2024).

The study is reported according to the 2025 CONsolidated Standards of Reporting Trials (CONSORT) guidelines. Between 12th January and 30th June 2023, 633 potential participants were screened, 239 were excluded commonly for: being ≥25 years (70) and not being sexually active in the past 3 months (61). Details of all exclusions are in Figure 1. Consequently, 394 participants were enrolled and randomized (197 per arm) and followed up until 11th June 2024 for a median time of 8.3 months (IQR 5.1−11.0).

#### 3.1.1 Intervention Delivery Per Arm

Overall, 328 (83.2%) participants attended at least one session of individualized health worker counselling (intervention = 168 [85.3%]; control = 160 [81.2%]), while 159 (80.7%) in the intervention arm attended at least one peer support session.

### 3.2 Participant Characteristics

Baseline characteristics were similar between arms (all *p*>0.05). Mean age was 19.4±2.4 years in the intervention arm and 19.4±2.3 in the control arm. Most had less than secondary education (intervention 82.7%; control 83.8%) and reported sex work as their main occupation (intervention 77.2%; control 80.7%). The majority (intervention 87.3%; control 86.3%) had high HIV risk perception, mainly due to concurrent sexual partners and unknown partner HIV status. Overall, the main sources of HIV‐related information were health workers (50.8%) and peers (26.4%), and 45.4% preferred oral PrEP for HIV prevention (Table 2). The mean creatinine clearance among PrEP initiators was 109 ± 26.9 mL/min.

**TABLE 2:** Baseline characteristics of 394 AGYW in an RCT assessing the effect of peer support on oral PrEP uptake and adherence in Kampala, Uganda, (2023−2024).

Characteristic	Intervention (*n* = 197)		Control (*n* = 197)	
	*n*	Col %	*n*	Col %
**Socio‐demographic characteristics**				
Participant age group at enrolment				
Adolescent girls (14−19 years)	106	53.8	100	50.8
Young women (20−24 years)	91	46.2	97	49.2
Education level				
Less than secondary	163	82.7	165	83.8
Secondary or higher	34	17.3	32	16.2
Marital status				
Single (never married)	158	80.2	171	86.8
Currently /previously married	39	19.8	26	13.2
Main occupation				
No job	9	4.6	13	6.6
Sex work	152	77.1	159	80.7
Hospitality/entertainment/other	36	18.3	25	12.7
**Reproductive health and behaviour risk**				
Using a reliable contraceptive	83	42.1	74	37.6
Total number of sexual partners in the past 3 months^a^				
<5 partners	59	30.4	62	31.6
5−9 partners	46	23.7	59	30.1
≥10 partners	89	45.9	75	38.3
Condom use with sexual partners in the past 3 months^a^				
Never or inconsistent	113	60.1	112	57.7
Consistent	77	39.9	82	42.3
Reported forced sex in the past 3 months	53	26.9	64	32.5
Travelled frequently in the past 3 months (≥3 nights/week)	138	70.0	139	70.6
Reported HIV status of sexual partner(s)				
HIV negative	36	18.3	35	17.8
Unknown or living with HIV	161	81.7	162	82.2
**HIV risk perception, information sources, prevention preference**				
Participant perceived herself at risk of HIV acquisition				
Yes	172	87.3	170	86.3
Most common source of HIV prevention information				
Community leaders	14	7.1	16	8.1
Healthcare providers	101	51.3	99	50.3
Peers	51	25.9	53	26.9
Social media	6	3.1	7	3.6
Electronic, print media	25	12.7	22	11.2
Preferred oral PrEP for HIV prevention				
Yes	86	43.7	93	47.2
**Intimate partner violence and substance use**				
Physical violence from sexual partners in the past 3 months^a^	66	34.4	65	33.7
Sexual violence from sexual partners in the past 3 months^a^	49	26.1	51	26.7
Use of drugs of addiction in the past 3 months	59	30.0	62	31.5

Abbreviations: AGYW, adolescent girls and young women; PrEP, pre‐exposure prophylaxis; RCT, randomized controlled trial.

^a^ *N* <394 due to data missing by error for (i) number of sexual partners (intervention = 3, control = 2), (ii) condom use with sexual partners (intervention = 7, control = 3), (iii) physical violence (intervention = 5, control = 4), (iv) sexual violence (intervention = 9, control = 6).

### 3.3 Primary Outcomes

#### 3.3.1 PrEP Uptake

Overall, 195 (49.5%) participants initiated PrEP: (intervention = 51.3%, control = 47.7%); (OR 1.15; 95% CI 0.77−1.71) (Table 3).

**TABLE 3:** Effect of peer support on PrEP uptake and adherence among 394 AGYW in an RCT, Kampala, Uganda (2023−2024).

PrEP outcome	Intervention arm	Control arm	OR (95% CI)	*p*‐value
PrEP uptake	101/197 (51.3%)	94/197 (47.7%)	1.15 (0.78−1.71)	0.481
Optimal adherence (85% pill count) at all PrEP refills	97/177 (54.8%)	53/134 (39.6%)	2.20 (1.23−3.95)	0.008
Optimal adherence (positive urine tenofovir assay) at all PrEP refills	97/177 (54.8%)	54/134 (40.3)	2.18 (1.22−3.91)	0.009

Abbreviations: AGYW, adolescent girls and young women; PrEP, pre‐exposure prophylaxis; RCT, randomized controlled trial.

#### 3.3.2 PrEP Adherence

Of 195 PrEP initiators, 163 (83.6%) (intervention = 45.1%, control = 38.5%) returned for at least one refill, contributing 311 refill visits (intervention = 177, control = 134). The average refill visits per participant were 2.1 (intervention) and 1.8 (control). Pill counts demonstrated strong agreement with urine tenofovir tests (*k* = 0.79; 95% CI 0.68−0.90) (Table 3).

Optimal adherence was observed at 150/311 (48.2%) refill visits by *pill count*; (intervention = 54.8%, control = 39.6%), (OR 2.20; 95% CI 1.23−3.95) and 151/311 (48.6%) by the *urine tenofovir assay* (intervention = 54.8%, control = 40.3%), (OR 2.18; 95% CI 1.22−3.91) (Table 3).

Sensitivity analysis excluding nine intervention‐arm participants who missed all peer sessions yielded similar effects (pill count OR 2.38; 95% CI 1.32−4.29; urine test OR 2.35; 95% CI 1.30−4.25). There was no significant intervention effect over time (chi^2^>3.84; *p*>0.05). After Bonferroni adjustment, PrEP uptake remained non‐significant (OR 1.15; 97.5% CI 0.73−1.81), while adherence remained statistically significant by pill count (OR 2.20; 97.5% CI 1.13−4.30) and urine assay (OR 2.18; 97.5% CI 1.12−4.26). Holm adjustment confirmed the Bonferroni‐adjusted results (uptake, *p* = 0.56) and (adherence, *p* = 0.016, *p* = 0.018).

#### 3.3.3 Other Analyses

The proportion included in the adherence analyses did not differ by study arm (*p* = 0.167) (Table S1). Adherence findings were robust when participants with no PrEP refills were classified as non‐adherent (Table S2).

The odds of optimal adherence by pill count and urine assay, respectively, were 79% and 72% higher with every additional intervention session attended (OR 1.79, 95% CI 1.26−2.55; *p* = 0.001) and (OR 1.72, 95% CI 1.20−2.49; *p* = 0.004).

Attendance of interventions sessions overall and by peer leader group are shown in Table S3 and Figure S1, respectively. AGYW facilitated by peer leaders on PrEP had higher odds of optimal adherence, though not significant (Table S4).

**FIGURE 2:**
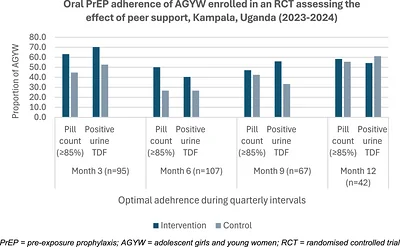
Optimal PrEP adherence by quarterly study visits among AGYW enrolled in an RCT assessing the effect of peer support on oral PrEP uptake and adherence, Kampala, Uganda (2023−2024).

Despite an intervention effect, optimal adherence remained generally low throughout but higher in the intervention arm (Figure 2).

### 3.4 Secondary Outcomes

#### 3.4.1 PrEP Persistence

**FIGURE 3:**
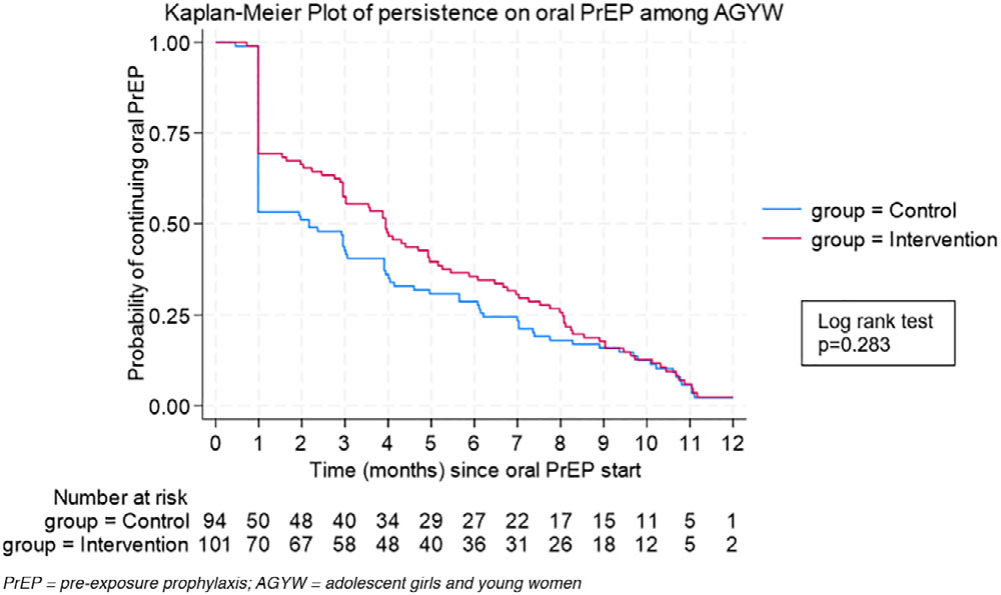
Kaplan−Meier plot of PrEP persistence among 195 AGYW who started PrEP in an RCT assessing the effect of peer support on oral PrEP uptake and adherence, Kampala, Uganda (2023−2024).

Overall, intervention‐arm participants had a higher PrEP continuation probability. The proportions still taking PrEP by month 6 (when implementation had been completed) were intervention (35.6%) and control (27.7%). By month 9, continuation probability was similar (20%), dropping to <5% in both arms by month 12. The median PrEP duration was 38 days (IQR 0–319 days); intervention (56 days), control (29 days). Continuation beyond 38 days was higher in the intervention arm (56.4% vs. 42.6%, *p* = 0.053). Discontinuation did not differ by arm (log‐rank: *p* = 0.283) (Figure 3). The intervention had no effect on PrEP persistence (HR 0.86; 95% CI 0.64−1.14, and the proportional hazards assumption was met (global *p* = 0.236).

#### 3.4.2 Contamination

Overall, 74 (45.4%) of the 163 participants with ≥1 refill (intervention = 81.1%, control = 18.9%) reported discussing the intervention with other AGYW, mainly focusing on the HIV risk reduction and PrEP education components. Exclusion of potentially contaminated control participants yielded similar results as the main adherence analysis (pill count OR 2.15, 95% CI 1.18–3.90; urine test OR 2.06, 95% CI 1.13−3.75).

#### 3.4.3 Study Completion

Completion of month 12 was 65.0% (intervention = 66%, control = 64%) with higher retention in the intervention arm at all visits (Figure S2). Baseline characteristics were similar between completers and non‐completers (Table S5).

#### 3.4.4 Intervention Cost‐Effectiveness

Delivering the 4‐month intervention cost $887 (average $9.24 per peer leader per session), of which 87.8% was peer leader allowances and the remainder was training costs. When using pill count as the adherence metric, the intervention averted an estimated 1.53 DALYs, resulting in an ICER of $581 per DALY averted. When using tenofovir testing as the adherence metric, the intervention averted 1.93 DALYs, yielding an ICER of $459 per DALY averted (Tables S6 and S7). Compared with a cost‐effectiveness threshold of $537, the intervention is cost‐effective when adherence is measured by urine tenofovir assays. Sensitivity analysis showed that the intervention would not be cost‐effective in settings where HIV incidence is <2.5% (Figure S3).

#### 3.4.5 HIV Acquisitions

We observed seven HIV acquisitions over 244.9 person‐years (incidence 2.86/100 person‐years; 95% CI 1.36–5.99). Five occurred in the intervention arm: two never initiated PrEP, two interrupted use and one had sub‐optimal adherence. Two occurred in the control arm: one non‐initiator and one with sub‐optimal adherence. All acquisitions were in 20‐ to 24‐year‐olds, mostly preferring longer‐acting PrEP options. Resistance testing in two cases detected no drug resistance mutations.

## 4 Discussion

This randomized trial evaluated a co‐created peer support intervention among AGYW at high risk of HIV acquisition delivered alongside health worker counselling with immediate drug‐level feedback. About half initiated PrEP. The intervention did not improve uptake, but significantly improved adherence, though overall adherence and persistence were low. It was cost‐effective when adherence was assessed using urine tenofovir testing, with greater cost‐effectiveness expected in higher‐incidence settings.

Several factors explain the null effect on PrEP uptake. The 1‐month delay in implementation likely missed participants contemplating PrEP initiation after health worker counselling. Unblinded health workers may have introduced performance bias by preferentially counselling control participants. Non‐random attrition among less motivated control‐arm participants may have biased effects towards the null, although baseline characteristics were similar between completers and non‐completers. Finally, high perceived HIV risk in both arms (>80%) and PrEP availability likely contributed to non‐differential uptake.

The improvement in adherence aligns with evidence from Kampala showing that peer‐delivered HIV prevention services improved adherence among AGYW [23]. Conversely, other peer‐delivered interventions either did not improve adherence among young transgender women in Uganda [44], and social media‐ or text message‐based peer support shows mixed effects, possibly due to participants opting out of interventions [45, 46]. Our results may reflect our model: 2‐ to 2.5‐h group sessions enabling in‐depth discussion and experience sharing, likely addressing information gaps and boosting motivation [47, 48]. Real‐time urine tenofovir feedback and counselling was provided in both arms and may have improved adherence overall; however, because it was implemented in both arms, higher adherence in the intervention arm likely reflects the peer support effect. Twice‐yearly injectable lenacapavir showed 100% efficacy in AGYW in South Africa and Uganda [49], but access remains uncertain [50], though generic production agreements may improve availability [51]. Given limited HIV funding and daily oral PrEP being the main option, peer support remains crucial in high‐incidence settings.

Despite improved adherence, overall persistence remained low, consistent with prior studies [11, 13] and reflecting waning effects on persistence after intervention delivery ended; hence the need for intensified early retention support. Peer support may delay but not prevent long‐term discontinuation; over half discontinued by month 6 and <5% remained by 12 months, likely reducing power to detect differences. Delayed PrEP discontinuation remains programmatically meaningful in high‐incidence settings. Additional influences such as reduced risk perception, partner disapproval and stigma may have contributed [12, 14, 52]. Phone reminders may have improved attendance and intervention dose, thereby inflating intervention effects. Information sharing leads to contamination, making study arms non‐comparable and affecting internal validity. Cross‐arm contamination may attenuate effects, although the sensitivity analysis suggested limited impact.

Using the point‐of‐care urine tenofovir testing allowed comparison with the standard pill count measure. Pill counts are inexpensive but time‐consuming and prone to pill dumping; hence may overestimate adherence. The urine assay is rapid, non‐invasive and validated in different populations [33, 53] prone to whitecoat dosing and assesses short‐term adherence; hence may overestimate adherence. Both measures showed substantial agreement with the urine assay, showing greater cost‐effectiveness, likely by classifying fewer control‐arm participants as consistently adherent. Seven HIV acquisitions occurred among AGYW not on PrEP at acquisition, consistent with prior studies [11, 54, 55]; all were older, mostly preferring long‐acting PrEP, underscoring the importance of expanding access to injectable lenacapavir.

This single‐site study limits generalizability but provides causal evidence of an intervention effect.

Saturday‐only, facility‐based delivery restricted access and did not fully address contextual barriers. High attrition reduces statistical power to detect intervention effects and yields imprecise estimates; however, over‐enrolment preserved statistical power for the primary analyses. Higher attrition in the control arm could have inflated intervention effects if differential, and participants with poorer adherence were more likely to be lost to follow‐up. However, attrition did not differ significantly between study arms. Recruiting peer leaders from existing PrEP programmes was feasible and supports task shifting strategies. Randomization enables causal inference, and the intervention is adaptable for community‐based delivery, mobile‐health approaches and lower‐intensity models, for example fewer peer leaders per session.

## 5 Conclusions

The co‐created peer‐led intervention improved PrEP adherence among AGYW, but not uptake and persistence. Studies replicating such an intervention could benefit from immediate implementation to motivate AGYW contemplating PrEP uptake and receive support to cope with side effects that are more intense in the first month. Early retention strategies are needed to improve PrEP persistence among initiators. Where affordable, using urine tenofovir testing is cost‐effective; however, intervention implementation is recommended in high HIV‐incidence settings, where short‐term PrEP use is beneficial. Sustainable HIV prevention, however, requires expanding access to long‐acting PrEP. Future research should assess whether immediate peer support after PrEP counselling improves uptake and identify scalable delivery models, including hybrid and digital approaches. Given low adherence and persistence, peer support should be evaluated alongside long‐acting PrEP. Implementation research is also needed to identify cost‐effective adherence monitoring and strategies that sustain early engagement.

## Author Contributions

YM: Lead author, designed the study, coordinated the study, contributed to data acquisition, analysis and interpretation, wrote the initial draft and revised versions of the manuscript. AA: Contributed to study design and guided the data analysis. KRK: Contributed to data analysis and made substantial scientific contributions to manuscript drafts. MS: Made substantial scientific contributions to manuscript drafts. BK: Made substantial scientific contributions to manuscript drafts. MG: Made substantial scientific contributions to manuscript drafts. KK: Supervised the first author and made substantial scientific contributions to manuscript drafts. AME: Made substantial scientific contributions to manuscript drafts and contributed to acquiring funding. ER: Contributed to study design, supervised the first author, contributed to suggestions for analytical methods and made substantial scientific contributions to manuscript drafts. All authors contributed to interpretation of study results and critically commented on all versions of the manuscript. They approved the final version of the manuscript. All authors attest that they meet the ICMJE criteria for authorship.

## Funding

This project was part of the second European & Developing Countries Clinical Trials Partnership (EDCTP2) programme supported by the European Union (CSA2020NoE‐3102). The Swedish Physicians Against AIDS Research Foundation (FOb2022‐0003) also supported the project. The funders had no role in the design, conduct, analysis and reporting of the trial.

## Conflicts of Interest

The authors have no conflicts of interest to declare.

## Disclaimer

The contents of this manuscript are the responsibility of the authors and do not necessarily state or reflect those of EDCTP.

## Open Science

The trial was registered at clinicaltrials.gov on 23/Aug/2022 under trial registration number NCT05516602 (https://clinicaltrials.gov/study/NCT05516602). The project protocol containing details of the trial and statistical analysis plans can be accessed from the corresponding author upon reasonable request. The de‐identified participant data and data dictionary can be accessed by researchers fulfilling requirements through the data sharing committee of the MRC/UVRI and LSHTM Uganda Research unit (datasharing@mrcuganda.org).

## Supporting information




**Supporting File**: jia270210‐sup‐0001‐SuppMat.zip

## Data Availability

The data that support the findings of this study are available on request from the corresponding author. The data are not publicly available due to privacy or ethical restrictions.
