# Supplementary material for: Effect of a Peer Support Intervention for Oral Pre‐Exposure Prophylaxis Uptake and Adherence Among Adolescent Girls and Young Women at Risk of HIV Acquisition in Kampala, Uganda: A Randomized Controlled Trial
Source: J Int AIDS Soc. 2026 Sep 21;29(9):e70210. doi: 10.1002/jia2.70210 (PMC13591225; doi:10.1002/jia2.70210)
Supplement: Supplementary file 1 [file JIA2-29-e70210-s001.zip › Supplemental Figures_S1_S2_S3_JIAS.docx]

Attendance of peer group sessions by AGYW in a randomised controlloed trial evaluarting peer support for oral PrEP uptake and adherence

Proportion of AGYW

| Monthly peer group support sessions | Peer leaders on ART | Peer leaders on PrEP |
| --- | --- | --- |
| Session 1 | 37.3737% | 46.9388% |
| Session 2 | 48.4848% | 66.3265% |
| Session 3 | 55.5556% | 70.4082% |
| Session 4 | 51.5152% | 73.4694% |

*AGYW = Adolescent girls and young women*

**Supplemental Figure S1: AGYW’s attendance of peer group support sessions by facilitating peer leaders during four months of intervention implementation**

*AGYW = Adolescent girls and young women*

**Supplemental Figure S2: Study completion rates, overall and by study visits among AGYW enrolled in an RCT assessing peer support for oral PrEP uptake and adherence**

HIV Incidence rates and corresponding costs per DALY averted of the peer support intervention

Cost per DALY averted

| HIV incidence rate | Cost per DALY averted | Cost effectiveness threshold |
| --- | --- | --- |
|  |  | 537 |
| 0.1% | 12183.2 | 537 |
| 0.5% | 2436.65 | 537 |
| 1% | 1218.32 | 537 |
| 1.5% | 812.215 | 537 |
| 1.99% | 612.223 | 537 |
| 2% | 609.162 | 537 |
| 2.5% | 487.329 | 537 |
| 3% | 406.108 | 537 |
| 3.5% | 348.092 | 537 |
| 4% | 304.581 | 537 |
| 4.5% | 270.738 | 537 |

*DALY = Disability adjusted life year*

**Supplemental Figure S3:** **Sensitivity analysis showing cost effectiveness of the peer support intervention at different HIV incidence rates**
