# Supplementary material for: Effect of a Peer Support Intervention for Oral Pre‐Exposure Prophylaxis Uptake and Adherence Among Adolescent Girls and Young Women at Risk of HIV Acquisition in Kampala, Uganda: A Randomized Controlled Trial
Source: J Int AIDS Soc. 2026 Sep 21;29(9):e70210. doi: 10.1002/jia2.70210 (PMC13591225; doi:10.1002/jia2.70210)
Supplement: Supplementary file 1 [file JIA2-29-e70210-s001.zip › Supplemental Tables_S1_S2_S3_S4_S5_S6_S7_JIAS.docx]

**Supplemental Table S1: Comparison by arm of AGYW included versus excluded from adherence analyses among 195 AGYW who started oral PrEP in an implementation project, Kampala, Uganda (2023-2024)**

| **Returned for ≥1 PrEP refill** | **Intervention arm**  n (%) | **Control arm**  n (%) | **P value** |
| --- | --- | --- | --- |
| **Yes (included)** | 88 (87.1) | 75 (79.8) | 0.167 |
| **No (excluded)** | 13 (12.9) | 19 (20.2) |  |
| **Total** | **101** | **94** |  |

*PrEP = pre-exposure prophylaxis; AGYW = adolescent girls and young women*

**Supplemental Table S2: Effect of peer support on PrEP adherence in a sensitivity analysis that classified AGYW with no PrEP refills as non-adherent in an oral PrEP implementation project, Kampala, Uganda (2023-2024)**

| **PrEP outcome** | **Intervention**  **arm** | **Control**  **arm** | **OR (95% CI)** | **P- value** |
| --- | --- | --- | --- | --- |
| Optimal adherence (85% pill count) at all PrEP refills | 97/181 (53.6%) | 53/135 (39.3%) | 2.06 (1.16-3.68) | 0.014 |
| Optimal adherence (positive urine tenofovir assay) at all PrEP refills | 97/181 (53.6%) | 54/135 (40.0%) | 2.03 (1.13-3.62) | 0.017 |

*PrEP = pre-exposure prophylaxis; AGYW = adolescent girls and young women*

**Supplemental Table S3: AGYW’s attendance of peer support sessions during four months of intervention implementation (Jan 2023 – Oct 2023)**

| **Number of sessions attended** | **Number of participants** | **Percentage** |
| --- | --- | --- |
| None | 39 | 19.8 |
| One | 29 | 14.7 |
| Two | 30 | 15.2 |
| Three | 43 | 21.8 |
| Four | 56 | 28.4 |

*AGYW = Adolescent girls and young women*

**Supplemental Table S4: PrEP adherence by peer leader group among AGYW who were assigned to the peer support intervention in an oral PrEP implementation project**

| **PrEP outcome**  **(n=177 participant visits)** | **Peer leaders using PrEP** | **Peer leaders using ART** | **OR (95% CI)** | **P- value** |
| --- | --- | --- | --- | --- |
| Optimal adherence by pill count | 65/108 (60.2%) | 38/69 (55.1%) | 1.26 (0.63-1.37) | 0.516 |
| Optimal adherence by urine tenofovir test | 62/108 (57.4%) | 35/69 (50.7%) | 1.24 (0.57-2.69) | 0.581 |

*PrEP = pre-exposure prophylaxis; AGYW = adolescent girls and young women*

**Supplemental Table S5: Baseline characteristics of 394 AGYW comparing those who completed versus did not complete follow up in an oral PrEP implementation project that assessed peer support for PrEP use (2023-2024)**

| **Characteristic** | **Categories** | **Study completed**  **(n=256)** | | **Study not completed**  **(n=138)** | | **p-value** |  |
| --- | --- | --- | --- | --- | --- | --- | --- |
|  |  | **n** | **Col %** | **n** | **Col %** |  |  |
| **Sociodemographic characteristics** | | | | | | |  |
| Participant’s age group at enrolment |  |  |  |  |  | 0.974 |  |
|  | Adolescents (14-19 years) | 134 | 52.3 | 72 | 52.8 |  |  |
|  | Young Women (20-24 years) | 122 | 47.7 | 66 | 47.2 |  |  |
| Education level of participant |  |  |  |  |  | 0.096 |  |
|  | Less than secondary | 219 | 85.6 | 109 | 79.0 |  |  |
|  | Secondary or higher | 37 | 14.4 | 29 | 21.0 |  |  |
| Marital status of participant |  |  |  |  |  | 0.284 |  |
|  | Single (never married) | 210 | 82.0 | 119 | 86.2 |  |  |
|  | Currently / Previously married | 46 | 18.0 | 19 | 13.8 |  |  |
| Number of biological children |  |  |  |  |  | 0.873 |  |
|  | None | 169 | 66.0 | 90 | 65.2 |  |  |
|  | One or more | 86 | 34.0 | 49 | 34.8 |  |  |
| **Reproductive health and sexual behaviour risk** | | | | | | |  |
| Use of any contraception in past 3 months |  |  |  |  |  | 0.062 |  |
|  | No | 29 | 11.3 | 25 | 18.1 |  |  |
|  | Yes | 227 | 88.7 | 113 | 81.9 |  |  |
| Number of sexual partners in past 3 months * |  |  |  |  |  | 0.084 |  |
|  | One partner | 25 | 9.8 | 5 | 3.7 |  |  |
|  | 2-9 partners | 122 | 47.6 | 174 | 55.2 |  |  |
|  | ≥10 partners | 109 | 42.6 | 55 | 41.0 |  |  |
| Level of condom use in past 3 months * |  |  |  |  |  | 0.991 |  |
|  | Inconsistent | 149 | 58.9 | 79 | 59.0 |  |  |
|  | Consistent | 104 | 41.1 | 55 | 41.0 |  |  |
| Reported sexual partner(s) HIV status |  |  |  |  |  | 0.971 |  |
|  | Unknown or living with HIV | 210 | 82.0 | 113 | 81.9 |  |  |
|  | HIV negative | 46 | 18.0 | 25 | 18.1 |  |  |
| STI diagnosis (chlamydia, gonorrhoea) * |  |  |  |  |  | 0.641 |  |
|  | No | 200 | 78.4 | 111 | 80.4 |  |  |
|  | Yes | 55 | 21.6 | 27 | 19.6 |  |  |
| Participants perceived herself at risk of HIV |  |  |  |  |  | 0.490 |  |
|  | No | 36 | 14.1 | 16 | 11.6 |  |  |
|  | Yes | 220 | 85.9 | 122 | 88.4 |  |  |
| **Mental health, IPV, substance use** | | | | | | |  |
| Depression severity (PHQ9 Tool) |  |  |  |  |  | 0.097 |  |
|  | None | 178 | 69.6 | 81 | 58.7 |  |  |
|  | Mild symptoms | 60 | 23.4 | 44 | 31.9 |  |  |
|  | Major depressive symptoms | 18 | 7.0 | 13 | 9.4 |  |  |
| Experienced any IPV in past 3 months * |  |  |  |  |  | 0.496 |  |
|  | No | 95 | 38.5 | 45 | 34.9 |  |  |
|  | Yes | 152 | 61.5 | 84 | 65.1 |  |  |
| Drug use in past 3 months |  |  |  |  |  | 0.586 |  |
|  | No | 175 | 68.4 | 98 | 71.0 |  |  |
|  | Yes | 81 | 31.6 | 40 | 29.0 |  |  |
| Alcohol use disorder screen test (AUDIT) |  |  |  |  |  | 0.594 |  |
|  | Low to moderate risk drinker | 215 | 84.0 | 113 | 81.9 |  |  |
|  | High risk drinker | 41 | 16.0 | 25 | 18.1 |  |  |

******* *N <394 due to data missing by error for (number of sexual partners (n=4), condom use (n=7), STI diagnosis (n=1), and data missing by design due to participants opting not to respond to IPV questions (n=18)*

*AGYW = Adolescent girls and young women; PrEP = pre-exposure prophylaxis; IPV = intimate partner violence; AUDIT = Alcohol use disorder identification test*

**Supplemental Table S6: Cost per Disability Adjusted Life Years (DALYs) averted by implementing the peer support intervention, considering pill count adherence**

| **Variables for calculating ICER** | **Intervention (n=79)** | **Control (n=75)** | **Total (N=154)** |
| --- | --- | --- | --- |
| **AGYW on PrEP** |  |  |  |
| optimal adherence (85% pill count) at all visits | 25 | 13 | 38 |
| suboptimal adherence (<85% pill count) at any visit | 54 | 62 | 116 |
| **Expected HIV acquisitions (%)** |  |  |  |
| AGYW with optimal adherence | 0.234 | 0.234 |  |
| AGYW with suboptimal adherence | 2.34 | 2.34 |  |
| **Expected HIV acquisitions (n)** |  |  |  |
| AGYW with optimal adherence | 0.0585 | 0.0303 | 0.0889 |
| AGYW with suboptimal adherence | 1.2636 | 1.4508 | 2.7144 |
| **Total expected HIV acquisitions** | 1.3221 | 1.4812 | 2.8033 |
| **Difference in HIV acquisitions** |  |  | 0.1591 |
| **DALYs per arm** | 12.6918 | 14.2193 |  |
| DALYs averted | | | 1.5275 |
| ICER = $(887/1.5275) per DALY averted | | | |
| **ICER = $581 per DALY averted** | | | |

*DALY = Disability adjusted life year; ICER = incremental cost-effectiveness ratio*

**Supplemental Table S7: Cost per Disability Adjusted Life Years (DALYs) averted by implementing the peer support intervention, considering urine tenofovir tests**

| **Variables for calculating ICER** | **Intervention (n=79)** | **Control (n=75)** | **Total (N=154)** |
| --- | --- | --- | --- |
| **AGYW on PrEP** |  |  |  |
| optimal adherence (positive urine TDF) at all visits | 25 | 11 | 36 |
| suboptimal adherence (negative urine TDF) at any visit | 54 | 64 | 118 |
| **Expected HIV acquisitions (%)** |  |  |  |
| AGYW with optimal adherence | 0.234 | 0.234 |  |
| AGYW with suboptimal adherence | 2.34 | 2.34 |  |
| **Expected HIV acquisitions (n)** |  |  |  |
| AGYW with optimal adherence | 0.0585 | 0.0257 | 0.0842 |
| AGYW with suboptimal adherence | 1.2636 | 1.4976 | 2.7612 |
| **Total expected acquisitions** | 1.3221 | 1.5233 | 2.8454 |
| **Difference in HIV acquisitions** |  |  | 0.2012 |
| **DALYs per arm** | 12.6918 | 14.6236 |  |
| DALYs averted | | | 1.9318 |
| ICER = $(887/1.9318) per DALY averted | | | |
| **ICER = $459 per DALY averted** | | | |

*DALY = Disability adjusted life year; ICER = incremental cost-effectiveness ratio*
